# YAP1-MAML2-Rearranged Poroid Squamous Cell Carcinoma (Squamoid Porocarcinoma) Presenting as a Primary Parotid Gland Tumor

**DOI:** 10.1007/s12105-020-01181-9

**Published:** 2020-06-05

**Authors:** Abbas Agaimy, Robert Stoehr, Lars Tögel, Arndt Hartmann, Thomas Cramer

**Affiliations:** 1 Institute of Pathology, University Hospital, Krankenhausstrasse 8-10, 91054 Erlangen, Germany; 2 Department of Otorhinolaryngology, Head and Neck Surgery, Bundeswehrkrankenhaus, Berlin, Germany

**Keywords:** Porocarcinoma, Poroma, YAP1, MAML2, Squamous cell carcinoma, Salivary glands, Parotid, Head and neck

## Abstract

Porocarcinoma (synonym: malignant eccrine poroma) is a rare aggressive carcinoma type with terminal sweat gland duct differentiation. The squamous variant of porocarcinoma is even less frequent and might be indistinguishable from conventional squamous cell carcinoma (SCC). We herein describe the first case of a carcinoma presenting as a primary parotid gland malignancy in a 24-year-old male without any other primary tumor. Total parotidectomy and neck dissection were performed followed by adjuvant chemoradiation. The patient remained alive and well 10 months after diagnosis. Histology showed keratinizing SCC infiltrating extensively the parotid gland with subtle poroid cell features. Oncogenic HPV infection was excluded by DNA-based testing. NGS analysis using the TruSight RNA fusion panel (Illumina) revealed a novel *YAP1-MAML2* gene fusion. This gene fusion was reported recently in a subset of cutaneous porocarcinoma and poroma. This case of poroid SCC (or squamoid porocarcinoma) adds to the differential diagnosis of SCC presenting as parotid gland tumor and highlights the value of molecular testing in cases with unusual presentation.

**Electronic supplementary material:**

The online version of this article (10.1007/s12105-020-01181-9) contains supplementary material, which is available to authorized users.

## Introduction

Salivary gland carcinomas are uncommon. They represent < 1% of all cancers and 5% of all head and neck cancers [1]. Mucoepidermoid carcinoma and acinic cell carcinoma are the main histological types encountered in pediatric and young adult patients, while other histological types are mainly diseases of adults and the elderly [2, 3]. The majority of squamous cell carcinomas (SCC) presenting within intraparotid lymph nodes represent metastatic SCC of the sun-damaged head and neck skin of the elderly [4]. Accordingly, any intraparotid SCC is considered metastatic until proven otherwise. We herein present a novel case of primary parotid gland carcinoma presenting in a 24-year-old man without other primary tumor. The tumor showed predominant squamous differentiation with subtle poroid features and a *YAP1-MAML2* gene fusion, detected by next generation sequencing. This rare variant might have been underecognized as conventional SCC or high-grade mucoepidermoid carcinoma in the past.

## Case History

A 24-year-old male without remarkable clinical history presented with a slowly growing left parotid swelling present for 6 months and associated with weight loss, night sweating and fever. An open surgical biopsy was obtained at an external center and interpreted as keratinizing poorly differentiated SCC. The patient was then referred to our ENT clinic for further management. Staging with computerized tomography (CT scan) of the head and neck and thorax confirmed the presence of a left parotid mass associated with slightly enlarged ipsilateral lymph nodes, but no other lesions were found. Total parotidectomy and ipsilateral neck dissection were performed. Postoperative whole body imaging including PET scan revealed no other primary neoplasm. Moreover, panendoscopy of the upper and lower aerodigestive tract (with biopsies and ipsilateral tonsillectomy) and dermatological consultation failed to reveal any other primary tumor. Post-operative adjuvant chemoradiation was recommended. During radiotherapy, swollen contralateral cervical nodes were detected and a right-sided neck dissection was performed, which revealed reactive lymphadenopathy, but no metastatic disease. Currently (10 months after surgery), the patient is doing well without evidence of locoregional recurrence or metastasis.

## Materials and Methods

The tissue specimen was fixed in formalin and processed routinely for histopathology. Immunohistochemistry (IHC) was performed on 3-µm sections cut from paraffin blocks using a fully automated system (“Benchmark XT System”, Ventana Medical Systems Inc, 1910 Innovation Park Drive, Tucson, Arizona, USA) and the following antibodies: CK7 (OV-TL, 1:1000, Biogenex), p63 (SSI6, 1: 100, DCS), CK5 (clone XM26, 1: 50, Zytomed), EMA (E29, 1:20, Dako), CEA (polyclonal, 1:400, Dako), CK19 (RCK108, 1:300, Dako), p16 (clone JC8, 1:100, Santa Cruz Biotechnology), TP53 (DO-7, 1:50, Dako) and anti-NUT antibody (clone C52B1, 1:45, Cell Signaling).

### Molecular Testing

DNA was extracted from formalin-fixed paraffin-embedded tumor tissue and tested for oncogenic Human Papillomavirus (HPV) infection using the methods described previously [5]. RNA was isolated from formalin-fixed paraffin embedded (FFPE) tissue sections using RNeasy FFPE Kit of Qiagen (Hilden, Germany) and quantified spectrophotometrically using NanoDrop-1000 (Waltham, United States). Molecular analysis was performed using the TruSight RNA Fusion panel (Illumina, Inc., San Diego, CA, USA) with 500 ng RNA as input according to the manufacturer`s protocol. Libraries were sequenced on a MiSeq (Illumina, Inc., San Diego, CA, USA) with > 3 million reads per case, and sequences were analyzed using the RNA-Seq Alignment workflow, version 2.0.1 (Illumina, Inc., San Diego, CA, USA). The Integrative Genomics Viewer (IGV), version 2.2.13 (Broad Institute, REF) was used for data visualization.

## Results

### Pathological Findings

**Fig. 1:**
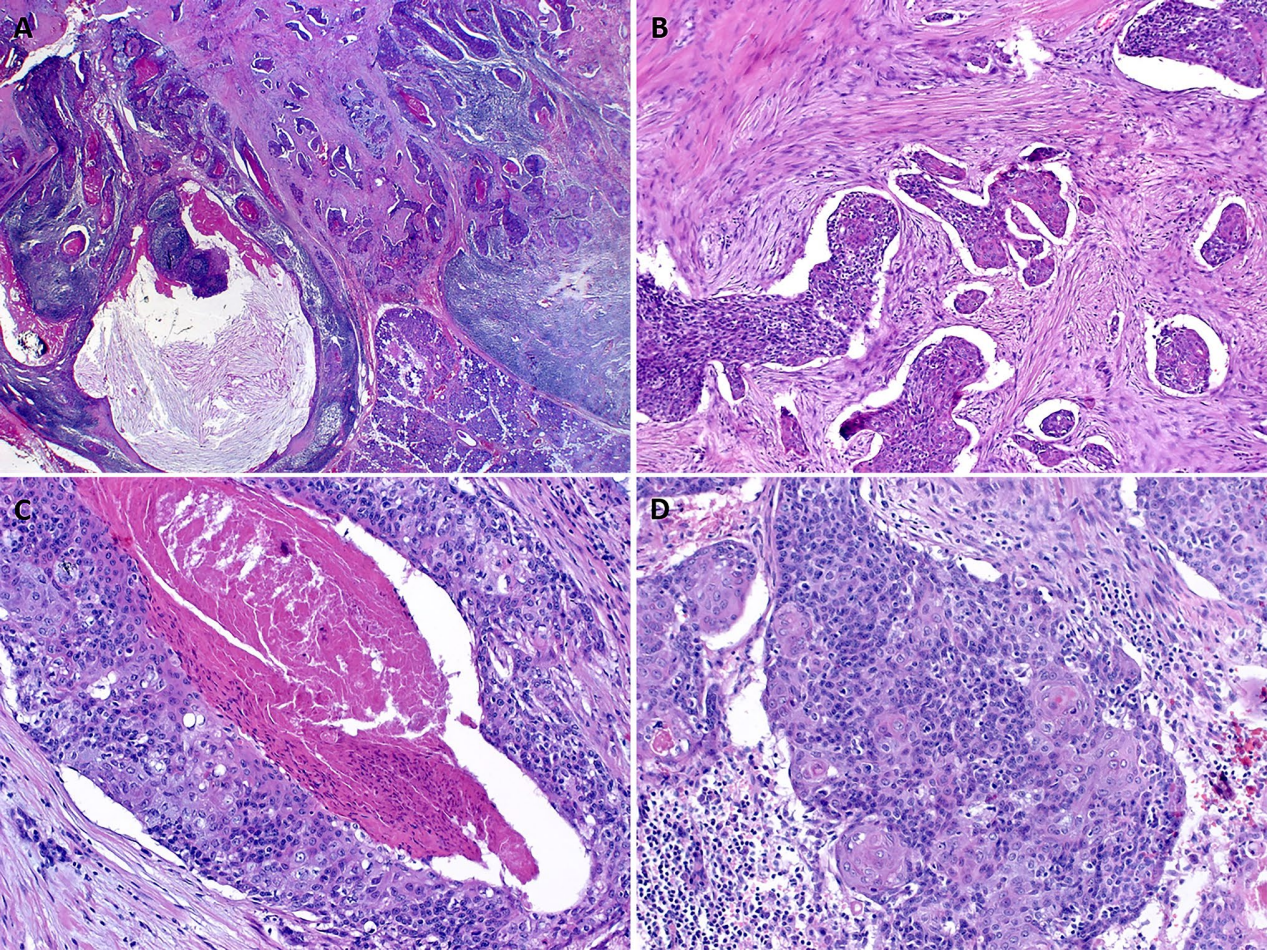
**a** Infiltrating squamous cell carcinoma is seen at low power, note prominent lymphoid stroma, variable cystic changes and keratinization. **b** The tumor cells are disposed into cohesive aggregates surrounded by desmoplastic stroma showing prominent retraction artefacts. **c** Abrupt adnexal-type keratinization and necrosis are seen at high power. **d** Smaller monomorphic blue-stained “poroid” cells might be mistaken for intermediate cells. Note follicle-like eosinophilic cuticle-type squamous cells amid the poroid cells

**Fig. 2:**
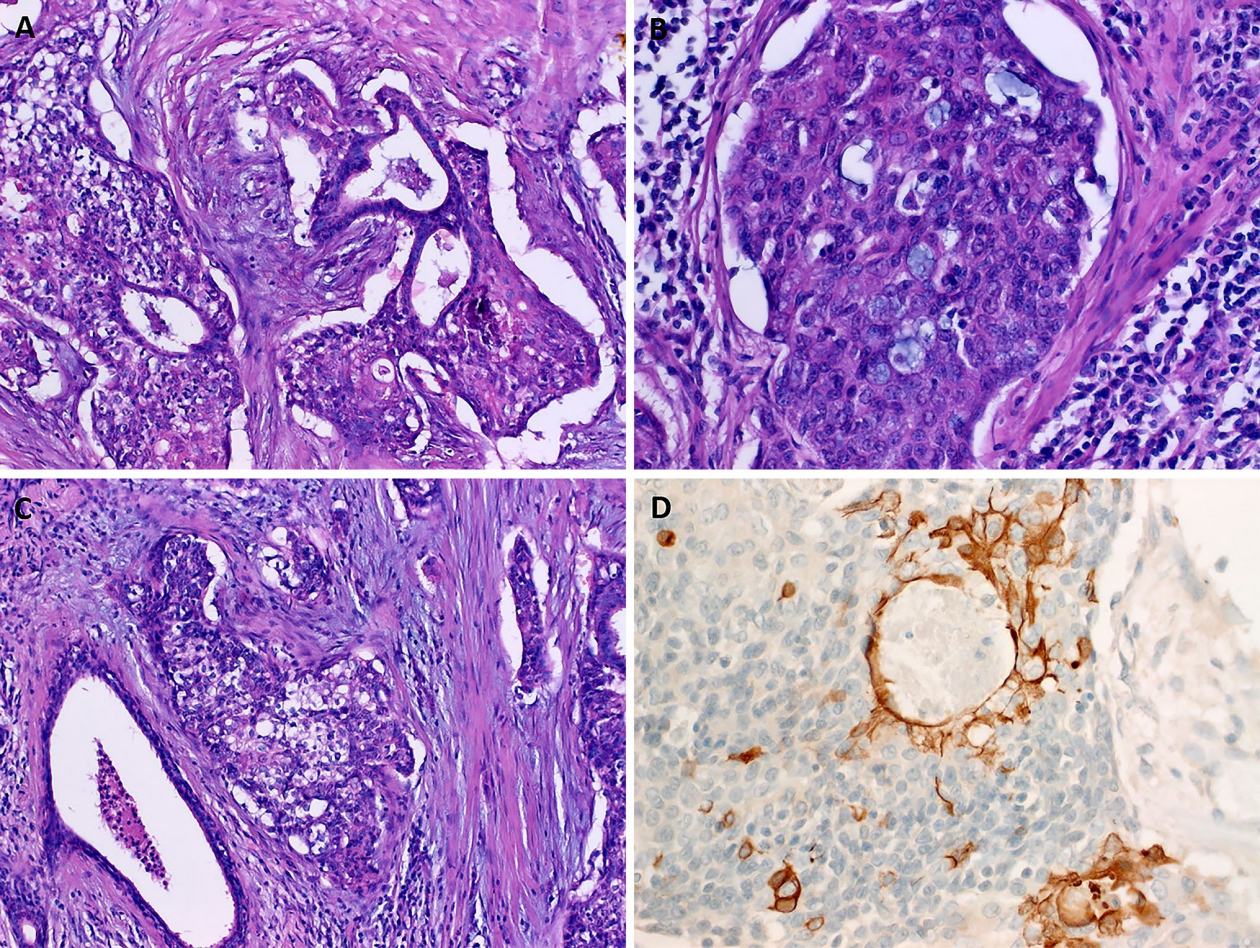
**a** Focal entrapment of native ducts mimicking epithelial-myoepithelial carcinoma. **b** small luminal spaces filled with mucinous material should not be mistaken for mucoepidermoid carcinoma. **c** Clear cell aggregates mimicking mucoepidermoid carcinoma are evident focally. **d** CK19 immunostaining highlights abortive and well-formed rare ductal spaces

Histological examination showed infiltrating carcinoma composed predominantly of highly atypical squamous cells with a variable degree of abrupt keratinization, focally forming prominent keratin-filed cystic spaces (Fig. 1a). A prominent lymphoid reaction was observed in the background stroma (Fig. 1a). The tumor cells were disposed into solid cohesive aggregates and nests surrounded by prominent retraction artifacts and stromal desmoplasia (Fig. 1b, c). In some areas, relatively smaller monomorphic cells with sparse cytoplasm were seen. These “poroid” cells were reminiscent of the intermediate cells of mucoepidermoid carcinoma (Fig. 1c, d). They were interrupted by bland-looking pale-eosinophilic squamous cell nests, consistent with the cuticle cells of poroma (Fig. 1c, d). Other findings included focal entrapment of native ducts (Fig. 2a), small intracellular lumina containing mucin droplets (Fig. 2b) and clear cell changes reminiscent of mucoepidermoid carcinoma (Fig. 2c). The tumor involved two intraparotid lymph nodes. One lymph node in the neck dissection specimen was involved as well (in total, 3 of 16 lymph nodes were positive). Perineural invasion was evident in the periphery of the gland.

Immunohistochemistry showed diffuse expression of p63, CK5 and TP53. P16 was expressed in < 70% of neoplastic cells. CK7 and the NUT antibody were negative. CK19, EMA and CEA highlighted a few foci of ductal differentiation (Fig. 2d).

### Molecular Findings

**Fig. 3:**
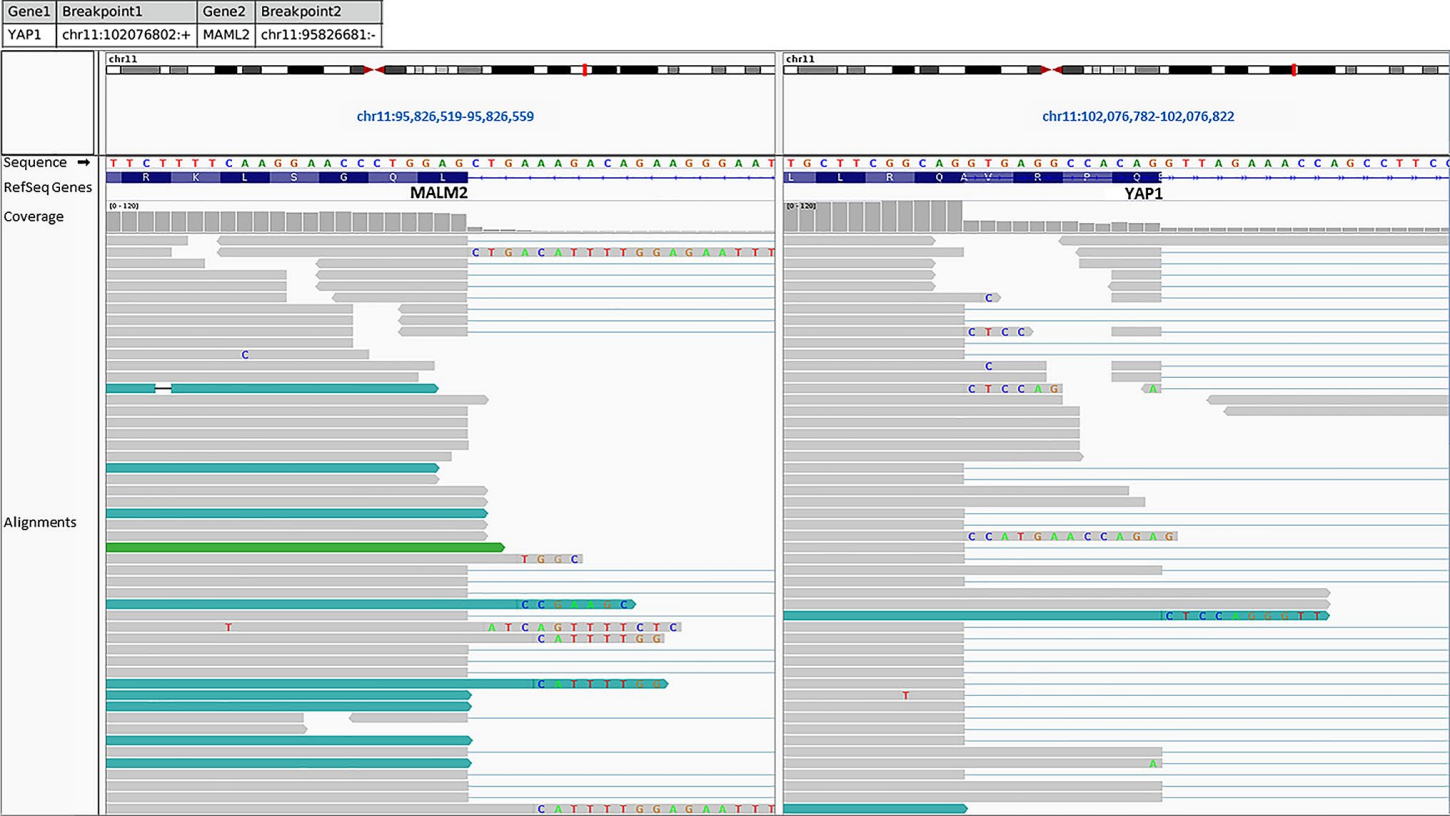
Integrated Genome Viewer (IGV) split-screen view of read alignments of the identified *YAP1-MALM2* Fusion event. Shown are the breakpoints in the *MALM2* locus (left) and the *YAP1* locus (right), respectively. Alignments whose mate pairs are mapped to the fusion sequence are colored turquoise

The DNA-based molecular testing for HPV revealed no evidence of oncogenic HPV infection. The NGS RNA sequencing analysis showed a *YAP1-MAML2* gene fusion, in which Exon 5 of *YAP1* is translocated presumably by intrachromosomal inversion to exon 2 of *MALM2* (Fig. 3). Both *YAP1* and *MAML2* are mapped to the long arm of chromosome 11 (at 11q22 and 11q21, respectively). Notably, there was no evidence of a *NUTM1* or *CTRC1/3-MAML2* gene rearrangements.

## Discussion

Primary salivary gland carcinomas with prominent squamous differentiation are rare. On the contrary, metastatic SCC presenting within the parotid gland is relatively common; the majority represent metastases from cutaneous head and neck SCC to intraparotid lymph nodes. In surgical series, SCC represented 6.6% of all parotid tumors and 41.5% of malignant cases [4]. In the files of the Hamburger Salivary Gland Registry, 75 of 10,944 salivary gland neoplasms were parotid metastases (37 localized within the parotid parenchyma and 38 within intraparotid nodes) [6]. Elderly men are predominantly affected at a mean age of 79.6 years [4]. The site of the primary tumor in metastatic cases was predominantly the head and neck skin, less frequently the pharynx and other primary head and neck locations, and rarely other extra-head and neck sites [4, 6]. Rare cases represented carcinomas of unknown primary [4].

A rare subset of high-grade salivary duct carcinoma and carcinoma ex pleomorphic adenoma with basal-like phenotype may display variable degree of squamous differentiation mimicking SCC and should be excluded by thorough sampling and other defined morphological features [7–9]. In the current case, there was no other tumor component, in particular no evidence of pre-existent pleomorphic adenoma or ductal differentiation. Moreover, RNA testing revealed no *PLAG1* or other gene fusions that would suggest origin from a pleomorphic adenoma.

NUT carcinoma may present rarely as primary salivary gland malignancy in the young age (adolescent and young adults) and it is therefore an important consideration [10]. However, squamous differentiation is usually limited in NUT carcinoma, represented by small foci of abrupt squamous differentiation in a background of poorly differentiated or undifferentiated neoplasm [10]. This possibility, however, is also excluded in the current case by negative NUT immunostaining and by absence of a *NUTM1* fusion at the molecular level. High-grade mucoepidermoid carcinoma is defined by *CTRC1/3-MAML2* fusion and histologically equivocal cases can be confirmed by molecular verification of the presence of the fusion [11].

If all of the differential diagnostic possibilities discussed above are reliably excluded, primary SCC of the parotid gland is vanishingly rare and is considered a diagnosis of exclusion [12]. In a recently published thorough literature review [13], the annual incidence of primary parotid SCC was in the range of 1.54 to 2.8 cases per million. However, upon critical reassessment, 30–86% of those cases initially reported as primary parotid SCC were found to represent metastases from skin or from upper aerodigestive tract SCC, highlighting an even much lower incidence than initially thought [13].

Thorough clinical workup supplemented by careful histopathological assessment is the only way to distinguish secondary metastatic SCC within the parotid from the vanishingly rare primary SCC. Distinguishing these two scenarios is of utmost clinical importance, given the significant differences in therapy and prognostication. However, there are currently no standard or reproducible criteria for confirming primary parotid SCC. Histological features proposed to be in favor of a primary parotid SCC include location within parotid parenchyma, absence of association with overlying skin, lack of mucin and intermediate cells and association with a duct or presence of squamous dysplasia in the ductal system [12, 13]. The etiology of primary SCC of the parotid gland is poorly understood, due to the rarity of the disease and to frequent confusion with metastatic SCC. Longstanding ductal obstruction due to lithiasis with subsequent squamous metaplasia and dysplasia has been proposed as explanation for primary SCC of the parotid gland [12].

The current case qualifies as SCC but showed unusual histological and clinical features leading to consideration of an alternative diagnosis: (1) the tumor presented at unusually young age (24 years in contrast to a mean age of 79 years for SCC of the parotid reported in the literature [4]; (2) it shows adnexal-type keratinization and variable other features reminiscent of adnexal-type differentiation; and (3) no other primary tumor could be found by thorough clinical investigation. This prompted us to perform NGS-based molecular testing to exclude unusual variants of translocation-related malignancies which revealed the *YAP1-MAML2* gene fusion.

MAML2 is a coactivator of transcription that is involved in the upregulation of the notch pathway target genes [14, 15]. *MAML2* is a well characterized fusion partner involved in the pathogenesis of salivary mucoepidermoid carcinoma, where it is fused to either *CRTC1* or *CRTC3* [15]. YAP1 on the other hand is a transcriptional coactivator involved in the regulation of the Hippo and Wnt pathways via TEAD-dependent mechanisms [16]. *YAP1-MAML2* gene rearrangements have been recently reported in rare nasopharyngeal carcinoma [17], all of metaplastic thymomas in one study [18] and in cell lines of diverse malignancies including glioblastoma, ovarian cancer and squamous cell carcinoma of the tongue [16, 19].

A recent study utilizing RNA sequencing and reverse transcription PCR analysis revealed a surprisingly high frequency of recurrent *YAP1-MAML2* fusions and the reciprocal *MAML2-YAP1* fusions in 71/104 (68%) poromas and in 1/11 (9%) porocarcinomas, respectively [20]. Poroma and porocarcinoma are rare skin adnexal neoplasms with features of terminal sweat duct differentiation [21]. As the malignant counterpart of poroma, porocarcinoma develops either from preexistent benign poroma or de novo [21]. The current case showed subtle features of conventional porocarcinoma including abortive ductal differentiation (highlighted by IHC) and adnexal-type keratinization associated with comedo-type necrosis [21]. Although squamous differentiation is considered uncommon in porocarcinoma [21], a predominantly squamoid variant has been proposed [22]. The predominance of squamous differentiation in the current case qualifies the tumor as squamoid porocarcinoma. The best terminology for the current case (SCC variant with poroid or acrosyringial differentiation versus porocarcinoma with extensive squamous cell differentiation) remains an issue of future studies, looking for molecular homologies or differences between the two entities.

The exact histogenetic origin of the tumor we are reporting herein is unclear. Given that some translocation-driven carcinoma variants of presumed ductal origin are shared by both the salivary glands and the skin adnexa including in particular mucoepidermoid carcinoma and secretory carcinoma [11, 15, 23], it is possible that this variant of salivary gland poroid SCC is of ductal origin as well, but this remains currently speculative.

As illustrated by the current case, age represents a strong clue to the differential diagnoses, in particular in unusually presenting neoplasms and is frequently suggestive of a genetically defined entity, hence warranting or justifying molecular testing. Indeed, all three possibilities in such a case of SCC-like malignancy of the salivary glands in a very young individual are translocation-driven: *NUTM1*-rearranged carcinoma, unusual variant of *MAML2*-related high-grade mucoepidermoid carcinoma and, based on the current case, *YAP1-MAML2*-driven squamous variant of porocarcinoma.

Distinguishing conventional cutaneous SCC from squamoid porocarcinoma of the skin is considered important in view of the different behavior of the two entities with regard to the higher risk for local recurrence, nodal involvement, systemic metastasis and overall mortality for porocarcinoma which is in contrast to the overall better outcome of conventional SCCs of the skin [22]. However, data comparing the outcome of SCC of the parotid gland and cutaneous porocarcinoma are lacking. While there is no need for fusion testing in cutaneous and other head and neck SCC metastatic to the parotid lymph nodes, molecular analysis of any putative primary SCC in the parotid gland, especially in the young, is recommended. Currently available sparse data precludes any reliable statement regarding the specificity of the *YAP1-MAML2* fusion as surrogate for “primary or metastatic porocarcinoma” versus “primary conventional SCC” of the parotid gland.

In summary, this is the first case of *YAP1-MAML2*-rerranaged poroid SCC (predominantly squamous porocarcinoma) presenting as a primary parotid gland malignancy in a young adult in the absence of another primary tumor. Inclusion of this entity in the differential diagnosis is mandatory to avoid misdiagnosis as conventional SCC and will help to delineate the prognostic and therapeutic implications of this uncommon putative entity.

## Electronic supplementary material

Below is the link to the electronic supplementary material.

Supplementary figure 1 (PNG 16 kb)

Supplementary figure 2 (PNG 16 kb)

Supplementary figure 3 (PNG 16 kb)

## References

[1] Foote FW, Frazell EL (1953). Tumors of the major salivary glands. Cancer.

[2] Brandwein-Gensler M, Bell D, Inagaki H, Katabi N, Leivo I, Seethala R, Triantafyllou A, El-Naggar AK, Chan JKC, Grandis JR, Takata T, Slootweg PJ (2017). Mucoepidermoid carcinoma. WHO classification of head and neck tumours.

[3] Simpson RHW, Chiosea S, Katabi N, Leivo I, Vielh P, Williams MD, El-Naggar AK, Chan JKC, Grandis JR, Takata T, Slootweg PJ (2017). Acinic cell carcinoma. WHO classification of head and neck tumours.

[4] Franzen A, Lieder A, Guenzel T, Buchali A (2019). The heterogenicity of parotid gland squamous cell carcinoma: a study of 49 patients. In Vivo.

[5] Agaimy A, Koch M, Lell M, Semrau S, Dudek W, Wachter DL, Knöll A, Iro H, Haller F, Hartmann A (2014). SMARCB1(INI1)-deficient sinonasal basaloid carcinoma: a novel member of the expanding family of SMARCB1-deficient neoplasms. Am J Surg Pathol.

[6] Seifert G, Hennings K, Caselitz J (1986). Metastatic tumors to the parotid and submandibular glands— analysis and differential diagnosis of 108 cases. Pathol Res Pract.

[7] Di Palma S, Simpson RH, Marchiò C, Skálová A, Ungari M, Sandison A, Whitaker S, Parry S, Reis-Filho JS (2012). Salivary duct carcinomas can be classified into luminal androgen receptor-positive, HER2 and basal-like phenotypes. Histopathology.

[8] Magaki SD, Bhuta S, Abemayor E, Nabili V, Sepahdari AR, Lai CK (2015). Carcinoma ex-pleomorphic adenoma of the parotid gland consisting of high-grade salivary duct carcinoma and keratinizing squamous cell carcinoma. Oral Surg Oral Med Oral Pathol Oral Radiol.

[9] Kusafuka K, Kawasaki T, Onitsuka T, Hamaguchi N, Morita K, Mukaigawa T, Nishiya Y, Kamijo T, Iida Y, Nakajima T, Sugino T (2020). Acantholytic squamous cell carcinoma and salivary duct carcinoma ex-pleomorphic adenoma of the submandibular gland: a report of two extremely rare cases with an immunohistochemical analysis. Head Neck Pathol.

[10] Agaimy A, Fonseca I, Martins C, Thway K, Barrette R, Harrington KJ, Hartmann A, French CA, Fisher C (2018). NUT carcinoma of the salivary glands: clinicopathologic and molecular analysis of 3 cases and a survey of NUT expression in salivary gland carcinomas. Am J Surg Pathol.

[11] Chenevert J, Barnes LE, Chiosea SI (2011). Mucoepidermoid carcinoma: a five-decade journey. Virchows Arch.

[12] Chiosea S, Hunt JL, Nagao T, Westra WH, El-Naggar AK, Chan JKC, Grandis JR, Takata T, Slootweg PJ (2017). Tumors of the salivary glands. Squamous cell carcinoma. WHO classification of head and neck tumours.

[13] Edafe O, Hughes B, Tsirevelou P, Goswamy J, Kumar R (2020). Understanding primary parotid squamous cell carcinoma—a systematic review. Surgeon.

[14] Kitagawa M (2016). Notch signalling in the nucleus: roles of Mastermind-like (MAML) transcriptional coactivators. J Biochem.

[15] Tonon G, Modi S, Wu L, Kubo A, Coxon AB, Komiya T (2003). t(11;19)(q21; p13) translocation in mucoepidermoid carcinoma creates a novel fusion product that disrupts a Notch signaling pathway. Nat Genet.

[16] Yuan Y, Park J, Feng A, Awasthi P, Wang Z, Chen Q, Iglesias-Bartolome R (2020). YAP1/TAZ-TEAD transcriptional networks maintain skin homeostasis by regulating cell proliferation and limiting KLF4 activity. Nat Commun.

[17] Valouev A, Weng Z, Sweeney RT, Varma S, Le QT, Kong C, Sidow A, West RB (2014). Discovery of recurrent structural variants in nasopharyngeal carcinoma. Genome Res.

[18] Vivero M, Davineni P, Nardi V, Chan JKC, Sholl LM (2020). Metaplastic thymoma: a distinctive thymic neoplasm characterized by YAP1-MAML2 gene fusions. Mod Pathol.

[19] Picco G, Chen ED, Alonso LG, Behan FM, Gonçalves E, Bignell G, Matchan A, Fu B, Banerjee R, Anderson E, Butler A, Benes CH, McDermott U, Dow D, Iorio F, Stronach E, Yang F, Yusa K, Saez-Rodriguez J, Garnett MJ (2019). Functional linkage of gene fusions to cancer cell fitness assessed by pharmacological and CRISPR-Cas9 screening. Nat Commun.

[20] Sekine S, Kiyono T, Ryo E, Ogawa R, Wakai S, Ichikawa H, Suzuki K, Arai S, Tsuta K, Ishida M, Sasajima Y, Goshima N, Yamazaki N, Mori T (2019). Recurrent YAP1-MAML2 and YAP1-NUTM1 fusions in poroma and porocarcinoma. J Clin Invest.

[21] Robson A, Greene J, Ansari N, Kim B, Seed PT, McKee PH, Calonje E (2001). Eccrine porocarcinoma (Malignant Eccrine Poroma): a clinicopathologic study of 69 cases. Am J Surg Pathol.

[22] Mahomed F, Blok J, Grayson W (2008). The squamous variant of eccrine porocarcinoma: a clinicopathological study of 21 cases. J Clin Pathol.

[23] Bishop JA, Taube JM, Su A, Binder SW, Kazakov DV, Michal M, Westra WH (2017). Secretory carcinoma of the skin harboring ETV6 gene fusions: a cutaneous analogue to secretory carcinomas of the breast and salivary glands. Am J Surg Pathol.

