# Supplementary figures and images for: YAP1-MAML2-Rearranged Poroid Squamous Cell Carcinoma (Squamoid Porocarcinoma) Presenting as a Primary Parotid Gland Tumor
Source: Head Neck Pathol. 2020 Jun 5;15(1):361–7. doi: 10.1007/s12105-020-01181-9 (PMC8010054; doi:10.1007/s12105-020-01181-9)

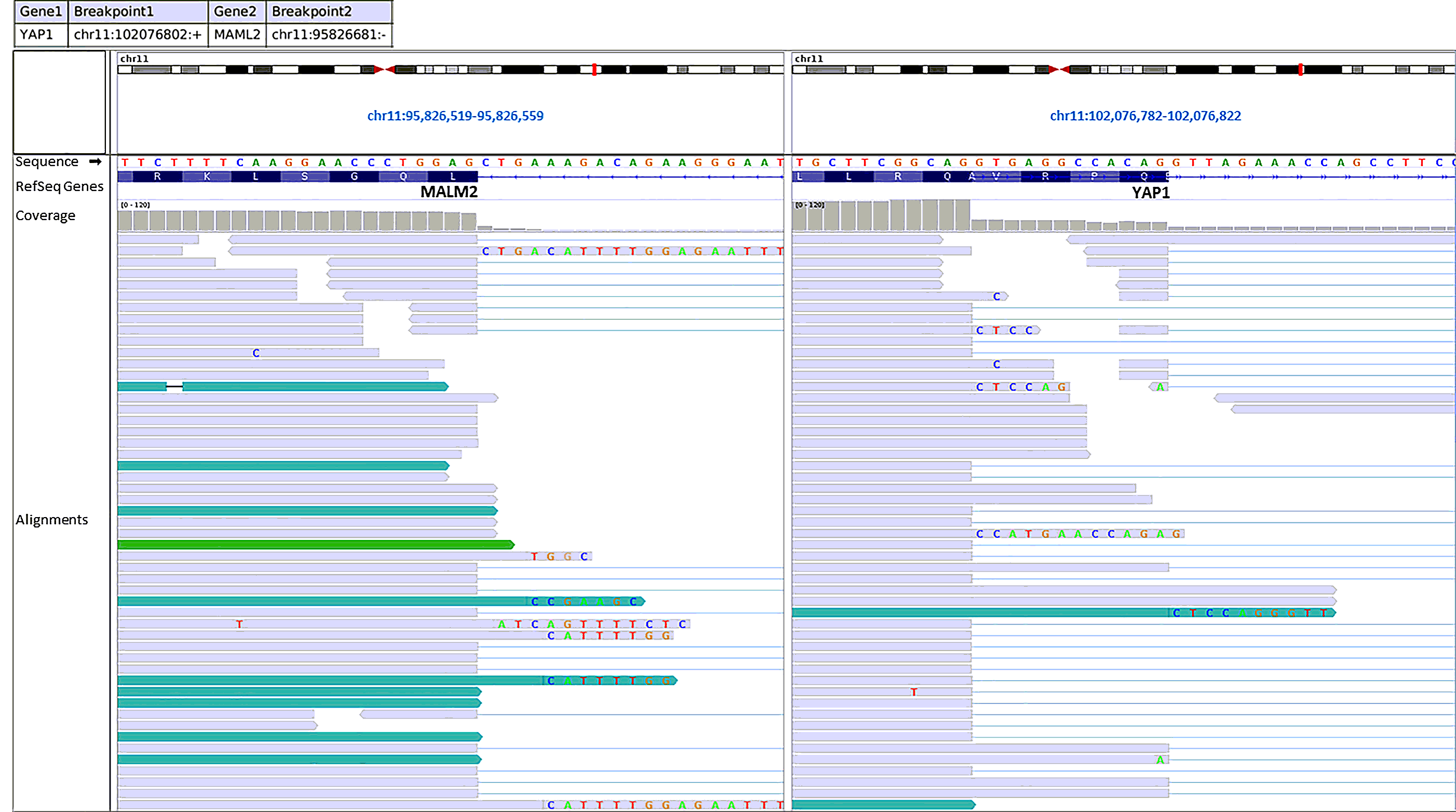

Supplement: Supplementary file 1 — Supplementary figure 1 (PNG 16 kb) [file 12105_2020_1181_MOESM1_ESM.png]

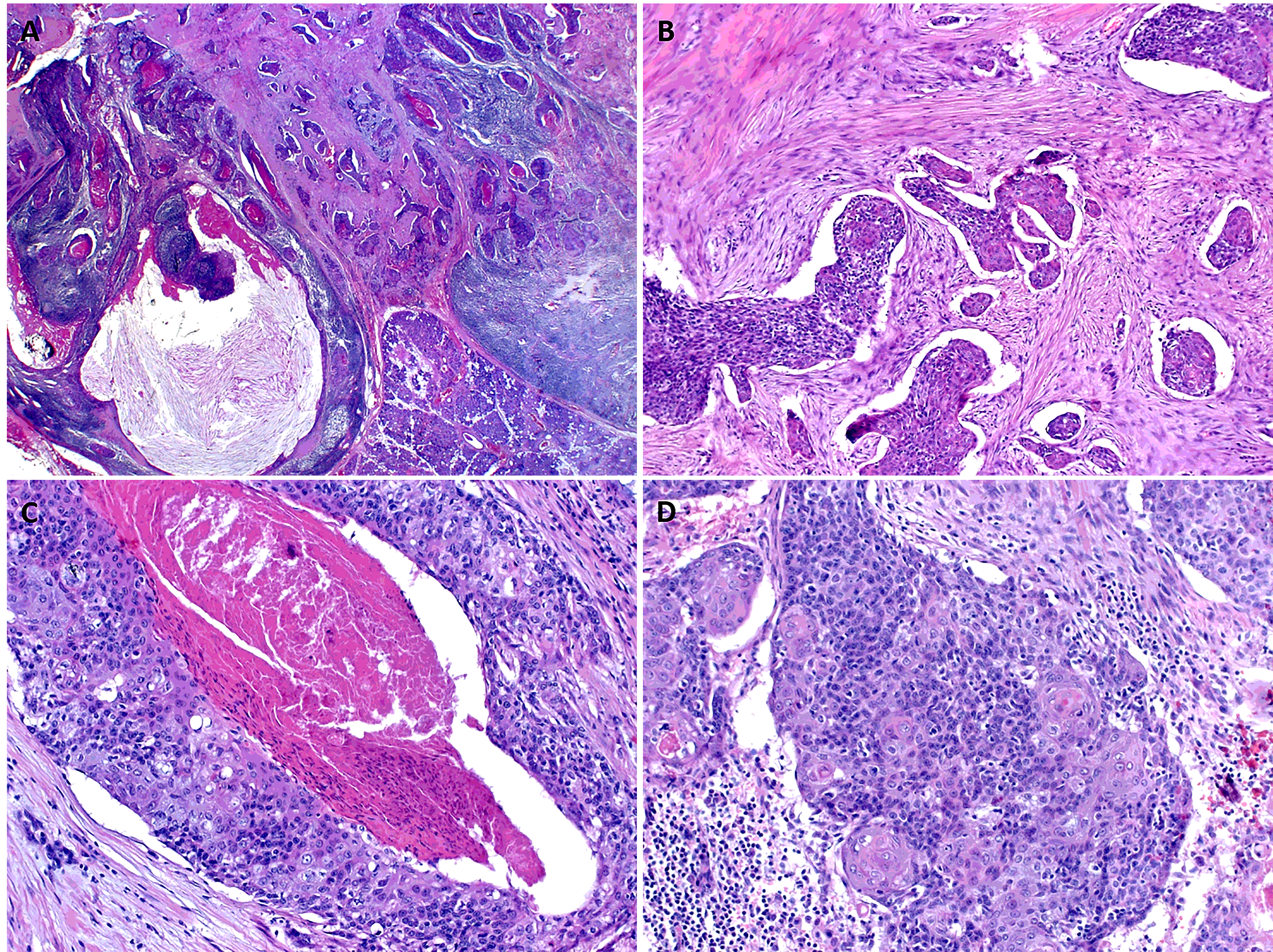

Supplement: Supplementary file 2 — Supplementary figure 2 (PNG 16 kb) [file 12105_2020_1181_MOESM2_ESM.png]

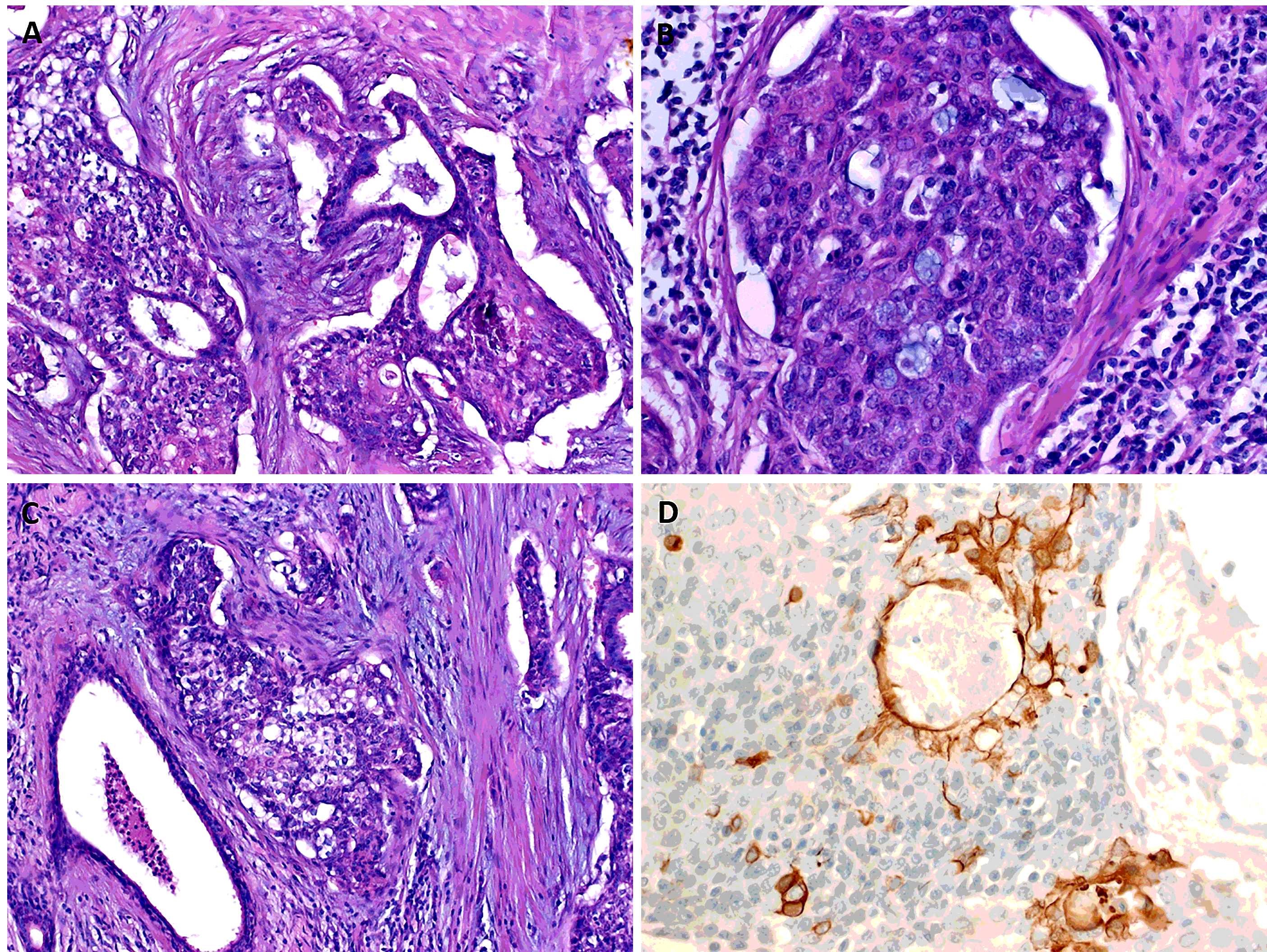

Supplement: Supplementary file 3 — Supplementary figure 3 (PNG 16 kb) [file 12105_2020_1181_MOESM3_ESM.png]
